# Broad T-Cell Receptor Repertoire in T-Lymphocytes Derived from Human Induced Pluripotent Stem Cells

**DOI:** 10.1371/journal.pone.0097335

**Published:** 2014-05-14

**Authors:** Chia-Wei Chang, Yi-Shin Lai, Lawrence S. Lamb, Tim M. Townes

**Affiliations:** 1 Department of Biochemistry and Molecular Genetics, University of Alabama at Birmingham, Schools of Medicine and Dentistry, Birmingham, Alabama, United States of America; 2 Department of Medicine, University of Alabama at Birmingham, Schools of Medicine and Dentistry, Birmingham, Alabama, United States of America; 3 Cell Therapy Lab, University of Alabama at Birmingham, Schools of Medicine and Dentistry, Birmingham, Alabama, United States of America; 4 UAB Stem Cell Institute, University of Alabama at Birmingham, Schools of Medicine and Dentistry, Birmingham, Alabama, United States of America; University of Minnesota, United States of America

## Abstract

Human induced pluripotent stem cells (hiPSCs) have enormous potential for the treatment of inherited and acquired disorders. Recently, antigen-specific T lymphocytes derived from hiPSCs have been reported. However, T lymphocyte populations with broad T cell receptor (TCR) diversity have not been generated. We report that hiPSCs derived from skin biopsy are capable of producing T lymphocyte populations with a broad TCR repertoire. In vitro T cell differentiation follows a similar developmental program as observed in vivo, indicated by sequential expression of CD7, intracellular CD3 and surface CD3. The γδ TCR locus is rearranged first and is followed by rearrangement of the αβ locus. Both γδ and αβ T cells display a diverse TCR repertoire. Upon activation, the cells express CD25, CD69, cytokines (TNF-α, IFN-γ, IL-2) and cytolytic proteins (Perforin and Granzyme-B). These results suggest that most, if not all, mechanisms required to generate functional T cells with a broad TCR repertoire are intact in our in vitro differentiation protocol. These data provide a foundation for production of patient-specific T cells for the treatment of acquired or inherited immune disorders and for cancer immunotherapy.

## Introduction

Mouse induced pluripotent stem cells (iPSCs) were first created by overexpression of Oct4, Sox2, Klf4 and c-Myc in somatic cells [1]. Subsequently, human iPSCs (hiPSCs) were produced by several groups with the same four factors or with Klf4 and c-Myc replaced by Nanog and Lin-28 [2]–[4]. Mouse and human iPSCs not only have similar morphologies, but also share two important features of embryonic stem cells (ESCs), pluripotency and self-renewal. The combination of these two properties plus the potential to grow unlimited numbers of cells that are isogenic to the somatic cell donor makes hiPSCs unprecedented in possible medical applications. The first “proof of the concept” application was performed in a humanized mouse model of sickle cell disease [5], [6]. Mouse iPSCs (miPSCs) derived from the humanized, sickle mice were corrected by gene replacement and differentiated into Sca-1 positive, c-Kit positive and SSEA-1 negative hematopoietic stem/progenitor cells (HSC/HPC). These cells were transplanted into humanized, sickle mouse recipients, and all red cell indices were restored to the normal range. Urine concentrating ability, which is a sensitive indicator of sickle pathology, was also restored. These results demonstrated that iPSC based gene/cell therapy can be used to cure an inherited disorder.

Correction of sickle cell disease suggested that disorders of other blood lineages might be possible with iPSC technology. Defects in T lymphocyte lineages result in many immunological disorders including severe combined immune deficiency (SCID) and autoimmunity. Gene correction or gene addition in patient-derived iPSCs followed by differentiation into transplantable hematopoietic progenitors or mature T cells would provide new approaches to treatment of these devastating disorders. Many groups have attempted to generate iPSC-derived HSC/HPC that are capable of differentiating into multiple blood cell lineages. The two most widely used methods form embryoid bodies (EBs) as an intermediate step or co-culture iPSCs with stromal cells to induce hematopoietic cell lineage specification [7]–[10]. Elisa et al [11] have demonstrated that *in vitro* generation of HSC/HPC by the EB method recapitulates in vivo hematopoietic cell development in which a group of cells (hemangioblasts) with both hematopoietic and vascular potential can be detected. Vodyanik et al [12] demonstrated that human ESCs (hESCs) co-cultured with a mouse bone marrow stromal cell line (OP9) could be used to produce CD34+/CD43+ cells, which are capable of differentiating into erythroid, myeloid and lymphoid lineages. Other studies demonstrated that hESCs co-cultured with stromal cells derived from the aorta-gonad-mesonephros (AGM) region of early mouse embryos or from fetal livers could also support production of CD34+ hematopoietic progenitors [9].

T-lymphocyte generation from mouse and human ESCs [10], [13] *in vitro* was reported several years ago, and recently several groups have demonstrated that T cells can be generated from human iPSCs [14]–[16]. However, these studies did not determine whether T cell specification followed the same stages of development that are observed in vivo or whether T cell specification from iPSC generated T cell populations with a broad TCR repertoire. Ultimately, the treatment of severe combined immune disorders (SCID) will require the generation of patient specific T lymphocyte populations with a broad TCR repertoire. In this paper, we describe the first example of highly diverse human T cells derived from iPSCs.

## Methods

### Human iPSCs Reprogramming and Characterization

Induced pluripotent stem cell lines were derived from primary skin cells obtained from skin biopsies. The biopsies were obtained under a protocol approved by the University of Alabama at Birmingham Institutional Review Board. The approved University of Alabama at Birmingham IRB Protocol number is F050914007, and the Informed Consent is approved through 10.09.2014. Participants signed the IRB approved consent form to indicate their consent. For iPSC induction, 5×10^4^ primary human keratinocytes were seeded on one well of a 6-well plate. On the following day, keratinocytes were transduced with 1 ml of virus supernatant and 1 ml of human keratinocyte medium containing polybrene at a final concentration of 4 ug/mL. The keratinocytes were spinfected at 800 g for 45 minutes (Day 1). The transduction procedure was repeated again the next day. On day 3, cells were changed to fresh human keratinocyte medium and cultured for two more days. On day 5, the keratinocytes were trypsinized and transferred to a 10 cm dish pre-seeded with mitomycin C-treated MEFs and cultured in human keratinocyte medium. On day 7, cells were changed to human ES medium and continuously cultured on the same dish for 3–4 weeks. ES medium was changed daily. Potential iPSC colonies were visible after 2–3 weeks. These colonies were individually picked and expanded on MEFs for analysis. To remove the integrated lentiviral and polycistronic sequences, iPSCs were infected with a Cre-expressing adenovirus (rAd-Cre-IE). Individual colonies were picked and Cre-mediated removal of floxed sequences was verified by PCR using primer set (gctaattcactcccaaagaagacaag and cttcagcaagccgagtcctg). After transferring hiPSCs to a 6-well plate for about 1 week, alkaline phosphatase staining (Vector Laboratories, Inc.) was performed. Pluripotent gene expression profiles were measured as follows. Total RNA was isolated from H1, PHK, hiPS-19 and hiPS-21 with Trizol reagent (Invitrogen), and 100 ng of total RNA was analyzed on an nCounter instrument (NanoString Technologies) as described by the manufacturer.

### Teratoma Formation

hiPSCs collected from 3 wells of a 6-well plate were suspended in a 100–150 µL volume of PBS and injected via a 21 G needle into the dorsal flanks of SCID mice. Tumors were recovered 4–8 weeks post-injection and processed for histological analysis.

Mouse protocols, techniques, anesthetics, analgesics, and number of mice used for the experiments described in this manuscript have been reviewed and approved by the Institutional Animal Care and Use Committee (IACUC) at the University of Alabama at Birmingham (UAB). All animal facilities at UAB are under the direction of full time veterinarians and are fully accredited by the American Association for Accreditation of Laboratory Animal Care. UAB complies with NIH policy on animal welfare (Letter of Assurance filed June, 1987 (3255-01)), the Animal Welfare Act and all other applicable federal, state and local laws.

### Generation of CD34+ cells and T cells with OP9 Co-culture

Cultures of hESCs or hiPSCs in one well of 6 well plate were treated as described by Ohnuki et al [17] with CTK solution to make small cell clumps. Cell clumps were then transferred to a 10 cm plate that was pre-seeded with OP9 cells in α-MEM-based medium containing 10% FBS, 1X penicillin/streptomycin and 100 µM mono-thioglycerol. The medium was changed every other day. On day 11 to 12, cells from one 10 cm plate were split onto two OP9 10 cm plates. One week after splitting (total of 18 days co-culture), cells were harvested by treating with dissociation solution (0.15% collagenase IV and 0.015% hyaluronidase in α-MEM medium) for about 30 minutes and followed by 0.25% trypsin for another 30 minutes. CD34+ cells were then purified on anti-CD34+ magnetic beads (MicroBead Kit; Miltenyi Biotec). For T cell differentiation, these CD34+ cells were plated on OP9-DL4 cells and cultured with α-MEM medium contains 20% FBS, 5 ng/ml hFlt3-L, 5 ng/ml hIL-7, and 10 ng/ml hSCF. The medium was changed every other day, and cells were transferred to new OP9-DL4 plates every 4 days. Four separate iPSC clones derived from fibroblasts of the same donor and two hESC lines (H1 and H9) generated CD3+, CD4+ and CD8+ T cells and NK cells (CD56+); the iPSC and hESC H1 results are presented below. All lines generated all of these T cell populations in every experiment (a total of 10 experiments; data not shown for 2 of the iPSC lines).

### TCRV_β_ CDR3 Region cDNA Sequencing

Total RNA was extracted from T cells generated *in vitro* from hiPS-21 derived CD34+ cells after 30 days of co-culture on OP9-DL4. First strand cDNA synthesis was primed with the specific primer TRBC-R4 (ctcaggcagtatctggagtcattgag) in the Superscript First-strand synthesis System (Invitrogen). To amplify the Vβ CDR3 region, PCR and nested PCR were performed with 17 different TRBV specific primers [18] and the reverse primer TRBC-R3 (tcaaacacagcgacctcgggtg). PCR products were cloned with the TOPO TA cloning kit (Invitrogen). Individual clones were picked and sequenced. Sequencing results were analyzed by Nucleotide Blast on the NCBI website.

### Generation of CD34+ Cells from Embryoid Bodies (EB)

We used an EB protocol described previously [7]. Briefly, H1 cells were treated with CTK solution [17] and manually disrupted in the plate into small clumps. Cells were transferred to low adhesive plates (Corning) and cultured with EB medium composed of stempro-34 medium (Invitrogen) supplemented with 1X penicillin/streptomycin, 10 ng/mL BMP-4 (R&D Systems), 2mM L-glutamine, 400 µM monothioglycerol, and 50 µg/mL ascorbic acid (Sigma). From this time point, cells were cultured in a 5% O_2_ incubator. On the next day, an equal amount of fresh EB medium with 10 ng/ml bFGF (Invitrogen) was added to the plate. After 3 days, cells were changed to EB medium with 10 ng/ml VEGF (Peprotech) and 5 ng/ml bFGF. After 3 or 4 days additional culture, CD34+ cells were harvested by disrupting the EBs with collagenase IV/trypsin and affinity purified (MicroBead Kit; Miltenyi Biotec).

### T cell Stimulation

*In vitro* derived T cells from hESCs and hiPSCs and primary T cells from human PBMC were stimulated by incubation with CD3/28 beads (Invitrogen) for 3 days before analysis by flow cytometry. For cytokines production assays, T cells were stimulated with 10 ng/ml PMA (Sigma) and 500 ng/ml ionomycin (Sigma) for 12 hours. GolgiStop and GolgiPlug (BD Bioscience) were also added at the same time.

### Flow Cytometry

Cells were harvested and washed before analysis with a LSRFortessa cell analyzer (BD Bioscience). For cell surface staining, PI was used to exclude dead cells. For intra-cellular staining, a Cytofix/Cytoperm Plus Brefeldin kit (BD Biosciences) or a Foxp3 Staining Buffer Set (eBioscience) was used to fix the cells. For T cell functional assays, Viability-506 (eBioscience) was used to exclude dead cells. Antibodies, which were used according to the manufacturers’ recommendations, were from BD Biosciences unless otherwise indicated. CD2-PE-Cy7 (clone L303.1), CD3 (V500, Percp-Cy5-5, PE-Cy5, clone UCHT1), CD4 (APC-Cy7, PE-Cy7, FITC, BV421, clone SK3), CD7-PE (eBioscience, clone 4H9), CD8 (V450, APC-Cy7, APC, clone SK1 and RPA-T8), CD25 (FITC, clone 2A3), CD31 (V450, clone WM59), CD34 (PE-Cy7, clone WM59), CD43 (PE, clone 1G10), CD45-APC-Cy7 (clone 2D1), CD56 (Percp-Cy5.5, APC, clone B159 and NCAM16.2), CD69 (FITC, PE-Cy7, clone L78 and FN50), CD309 (647, clone 89106), IL2-PE (clone MQ1-17H12), IFN-γ-V450 (clone B27), NKG2D-PE (clone 1D11), TCR-αβ (FITC, PE, clone T10B9.1A-31), TCR-γδ (FITC, PE, BV421, PE-CF594, clone B1, 11F2), TCR-V_δ_1-FITC (Fisher Scientific, Clone TS8.2), TCR-V_δ_2-PE (clone B6), TCR-V_γ_9-FITC (clone B3), TNF-α-PE-Cy7 (clone MAB11), TRA1-85-Fluorescein (R&D, clone TRA-1-85), Beta Mark TCR Repertoire Kit (Beckman Coulter).

### Colony Forming Cell (CFC) Assay

One hundred thousand, affinity-purified CD34+ cells derived from hESCs and hiPSCs were used in CFC assays. Cells were plated in MethoCult H4434 Classic (Stemcell Technologies) according to the manufacturer’s instructions. After two weeks of incubation, colonies were identified by morphology. All of our hiPSC and hESC lines generated erythroid and myeloid colonies including BFU-E, CFU-E, CFU-G, CFU-M, CFU-GM and CFU-GEMM.

### Vector Construction

To make the OSKM vector, polycistronic OSK [19] DNA sequences were PCR amplified from the pKP332 plasmid with primers (pDL171-F: tgggtggagactgaagttag and MuI-hKlf4-R: acgcgtaaaatgcctctt catgtgtaaggc). Human c-Myc cDNA was PCR amplified with primers (MluI-E2A-hmyc-F: attttacgcgtcagtgtactaattatgctctcttgaaattggctggagatgttgagagcaacccaggtcccatgcccctcaacgttagcttc and MluI-hmyc-R: acgcatacgcgtatttaaatttacgcacaagagttccgtagc). After digestion with MluI, c-Myc cDNA was ligated into the MluI site in the OSK plasmid. The OSKM polycistronic cDNA sequence was confirmed, and OSKM cDNA was digested with SwaI and cloned into the SwaI site of lentiviral vector pDL171. The Lenti-hDL4-mCherry plasmid was constructed by cloning a PCR-amplified human DL4 cDNA (openbiosystem), an IRES fragment (openbiosystem) and mCherry cDNA into the pDL171 vector. PCR reactions were performed using PrimeStar polymerase (Takara). All of the oligos used in this study were synthesized by Integrated DNA Technologies (IDT).

### Cell Culture

ESC and iPSC cells were cultured on mitomycin C-treated murine embryonic fibroblasts (MEFs, derived from E14.5 CF-1 embryos) in ES cell media consisting of DMEM F-12 supplemented with 1X non-essential amino acids, 1X penicillin-streptomycin, 1X L-glutamine (all from Mediatech), 20% knock serum replacement (Invitrogen), 2-ME (Sigma) and 5 ng/ml bFGF (Invitrogen). Human primary keratinocytes were cultured in DermaLife K Medium Complete Kit (LifeLine Cell Technology). OP9 cells were purchased from ATCC and grown in α-MEM medium with 20% FBS and penicillin-streptomycin. OP9-DL4 cells were established by transducing OP9 cells with lenti-hDL4-mcherry virus. The human embryonic stem cell line H1 (hESC H1) was obtained from the WiCell Research Institute. Induced pluripotent stem cell lines were derived from primary skin cells obtained from skin biopsies as described above under ‘Human iPSCs reprogramming and characterization’.

### Virus Production

For preparation of lentivirus, 10 ug of the lentiviral vector, 2.5 ug of the envelope plasmid (pMDG), and 7.5 ug of the packaging plasmid (pCMBVdR8.9.1) were co-transfected into 5×10^6^ 293 T cells by Fugene 6 (Roche or Promega). Virus-containing supernatant was collected 2 days after transfection, and passed through a 0.45 µm filter.

## Results

**Figure 1:**
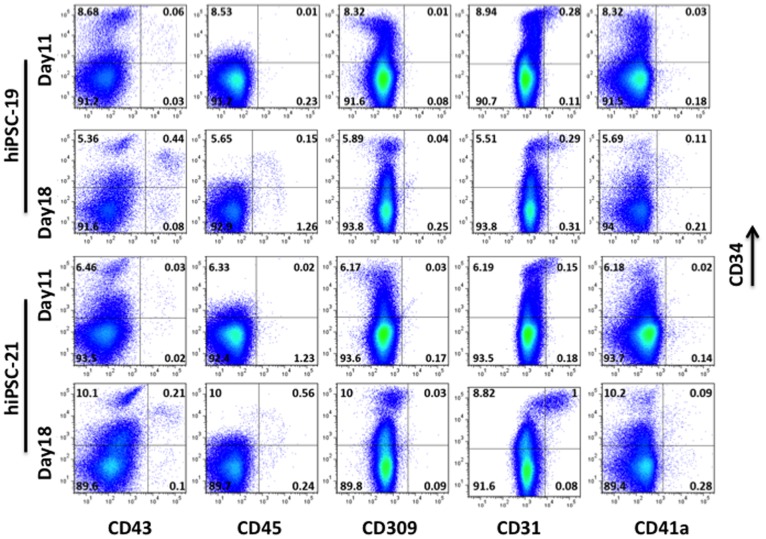
In vitro derivation of CD34+ HSC/HPC from Human iPSC. Human iPSC (hiPSC-19 and hiPSC-21; prepared as illustrated in Figure 1) were cultured on OP9 feeders for 11 days. The cultures were harvested, and samples were analyzed by FACS (Day11 cells). All remaining cells were transferred to fresh OP9 plates and cultured for an additional 7 days. These cells were subsequently harvested and analyzed by FACS (Day18 cells).

Human iPSCs were derived by transducing skin cells with a modified version of our “Hit and Run” lentiviral vector [19] carrying Oct4, Sox2, Klf4 and c-Myc cDNAs driven by the EF-1α promoter. After removal of the reprogramming factors by infection with adenovirus expressing Cre recombinase (Figure S1), pluripotent hiPSCs were co-cultured with OP-9 stromal cells to produce hematopoietic zone (HZ)-like structures. These endothelium-lined cell clumps have been described previously [9], [13], [20] when human hematopoietic progenitors were co-cultured on OP-9 feeders. HZ-like structures were observed at approximately 7 days in our culture conditions. Therefore, we established a 2-step method to expand HZs. hiPSCs were cultured on OP9 for 11 days, and a sample of these cells was analyzed by FACS (Day 11 cells). All remaining cells were transferred to fresh OP9 plates and cultured for an additional 7 days in which HZs expanded. These cells were harvested and analyzed by FACS (Day 18 cells). This two-step protocol resulted in consistent production of CD34/CD43 double positive cells from both hiPSCs (Figure 1) and hESCs (Figure S2). The fraction of CD34/CD43 double positive cells derived from both hiPSC lines that we analyzed (hiPSC-19 and hiPSC-21; Figure 1) increased approximately 7-fold from Day11 to Day18. The fraction of CD34/CD45 double positive cells derived from both hiPSC lines also increased significantly during this time frame (Figure 1). Therefore, we affinity-purified CD34+ cells generated from hiPSCs and control hESCs at Day18 under these conditions and analyzed the cells in colony forming cell (CFC) assays. BFU-E, CFU-E and CFU-GM colonies were observed from both groups (data not shown). These results demonstrated that CD34+ cells derived by this protocol were capable of differentiating into multiple myeloid lineages, and, therefore, might represent progenitors capable of both myeloid and lymphoid development.

**Figure 2:**
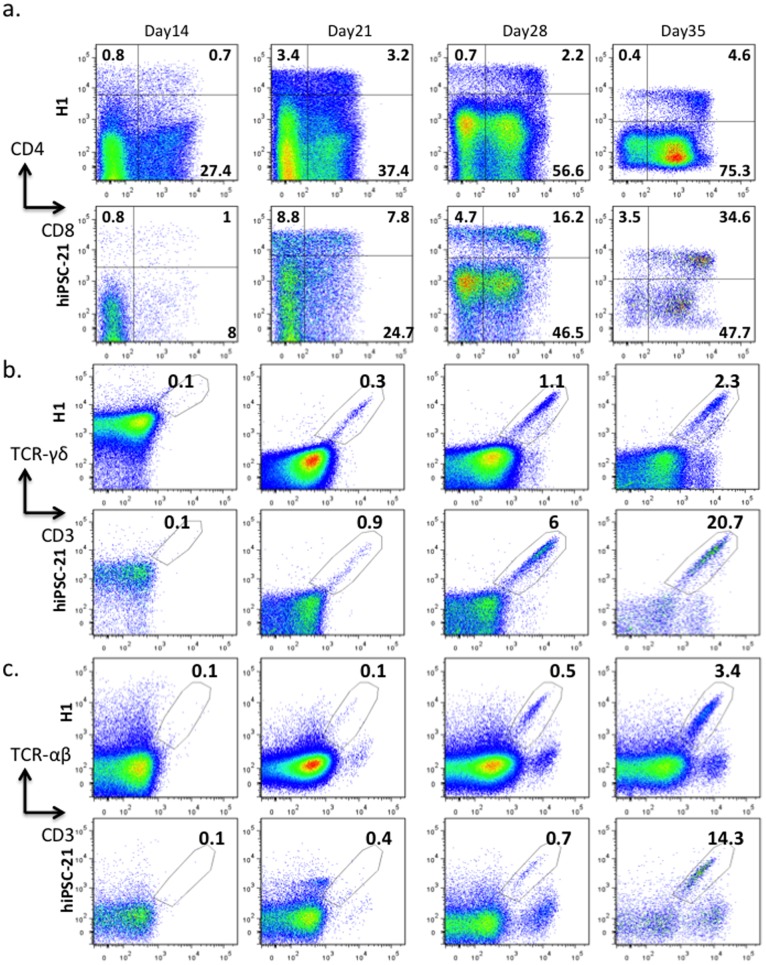
Generation of T lymphocytes from hiPSC and hESC (H1). Human iPSC- and hESC-derived CD34+ cells were purified on anti-CD34+ magnetic beads after a two-step, 18-day co-culture with OP9 cells and subsequently plated on OP9-DL4 stromal cells. After 14, 21, 28, and 35 days of co-culture, the cells were analyzed for the indicated T cell markers. **a**, CD4, CD8. **b**,TCR-γδ and surface CD3. **c**,TCR-αβ and surface CD3. Similar data for hiPSC-19 are illustrated in Figure S10).

Subsequently, we differentiated hiPSCs or hESCs (H1) for 18 days under the conditions described above, affinity-purified CD34+ cells, and then plated these cells on OP9-DL4 stromal cells [21]. To monitor T lineage commitment and T cell maturation, cells were collected from plates and analyzed for lymphocyte cell markers at day 7, 14, 21, 28 and 35 (Figure 2 and Figures S3–S9). CD7 and CD2 are early T cell markers [22], [23]. Both of these markers were detected as early as Day 7 (Figure S4). As the number of CD34 expressing cells decreased during extended culture, the number of CD1a, CD7 and CD2 expressing cells increased (Figures S3–S6). More than 99% of the population was CD7+ and more than 80% of the population was CD2+ at Day 35 (Figure S3). During normal T cell development in vivo, CD3 antigens are first detected as cytoplasmic proteins. As T cells mature, CD3 migrates to the cell surface [24]. T cells generated in our *in vitro* cultures mimicked this progression; expression of cytoplasmic CD3 (CD3cyt) was observed at Day14 (Figure S7a), and distinct surface CD3+ populations were not detected until Day21 (Figure S7b). The CD3cyt population was always under-represented in hESCs compared to hiPSCs. On the other hand, hESCs produced more NK cells (Figure S7b). Interestingly, we had to adjust the cell density in these cultures to prevent NK cells from killing the stromal cells and inhibiting further development. Slightly more CD4+ cells than CD8+ cells emerged at Day7 (Figure S8). At Day14, both CD4+ and CD8+ cells were detected (Figure 2 and Figure S8). After Day14, CD4+CD8− populations were always under-represented compared to CD4−CD8+. At Day35, only a few CD4+CD8− cells could be detected in the cultures; more than half of the cells in the entire population were CD4−CD8+ in both hiPSCs and hESCs-derived cultures (Figure 2 and Figure S8). The relative absence of CD4+CD8− cells derived from hiPSC, compared to CD4+CD8− cells derived from mobilized peripheral blood CD34+ cells (Figure S9), is similar to observations by others in T cell cultures derived from murine iPSCs and ESCs [10], [25].

Expression of T cell receptors (TCR) is another hallmark of T cell development. In our system, TCR-γδ positive cells are detected as early as Day 14 (Figure 2b, Figure S10a). At Day 21, a robust TCR-γδ population was observed with the three cell lines that we tested. In vivo, TCR-αβ positive cells mature later than the TCR-γδ population [26]–[28]. We observed this same developmental pattern in our *in vitro* cultures (Figure 2c and Figure S10b). TCR-αβ cells emerged about one week later than TCR-γδ cells, and the αβ populations were smaller than the γδ populations. Therefore, our *in vitro* differentiation system mimics human T cell development in vivo. In these *in vitro* conditions, populations of mature T cells that express CD3, CD4, CD8, TCR-γδ and TCR-αβ are consistently generated from hiPSCs (Figure 2 and Figs. S3–S11).

**Figure 3:**
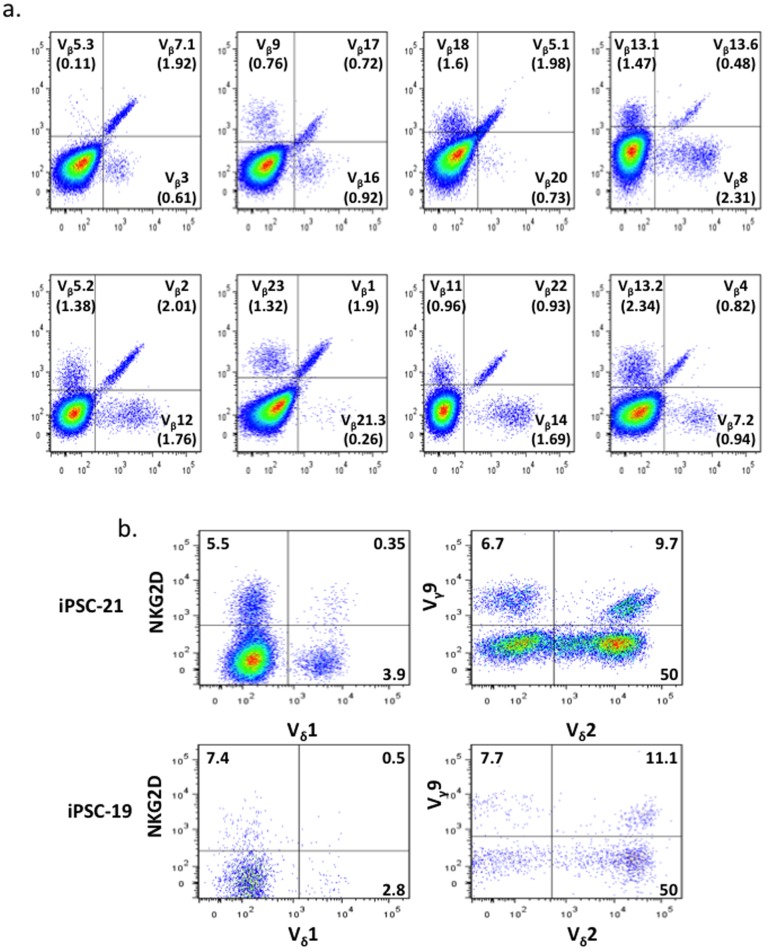
T cell receptor typing. CD34+ cells were affinity purified from Day18 cultures as described in the text and cultured on OP9-DL4. After 33 days of co-culture, the cells were analyzed by FACS. **a**, T cells derived from hiPSC-21 were typed for TCRV_β_ with the Beta Mark TCR Repertoire Kit from Beckman Coulter. The kit contains eight vials. Each vial contains 3 monoclonal antibodies that recognize 3 different V_β_ chains. The antibodies were conjugated with FITC, PE or FITC plus PE. A total 24 different mAbs in this kit can detect 24 V_β_ chains (belonging to 19 different families). The cells were gated on the CD3+ populations. **b**, TCRV_γ_, TCRV_δ_ and NKG2D expression were examined in T cells derived from hiPSC-19 and hiPSC-21. The data were gated on CD3+ and TCR-γδ+ populations.

The efficient recognition of an enormous set of foreign peptides by the T cell receptor (TCR) is essential for cell-mediated immunity. In the thymus, T cell precursors undergo TCR gene rearrangements that produce highly diverse junctions of V, (D), and J gene segments together with the addition of N nucleotides. To determine whether a diverse TCR repertoire is generated in our *in vitro* differentiation system, V_β_ typing was performed by flow cytometry. Human iPSCs derived T cells expressed all the V_β_ segments that we tested (19 of 25) [29] (Figure 3a), although the percentages are different from primary human peripheral T cells (Figure S12). We consistently detect a large γδ T cell population in our culture system (Figure 2 and Figure S10). In the CD3+ gated γδ T cell population, about 50% were V_δ_2+ and 3% were V_δ_1+ (Figure 3b). In vivo, V_δ_2V_γ_9 is the major subtype of peripheral recirculating γδ T cells [30]. In the two hiPSCs lines we tested, approximately 10% of V_δ_2 cells were V_δ_2V_γ_9. Interestingly, NKG2D was detected in our T cell populations. This receptor is important for γδ T cells to recognize stress-induced antigens on tumor cells [31].

Generation of CDR3 diversity in the T cell receptor is a critical step in the development of a functional immune system. Therefore, we analyzed the CDR3 region of TCR-β chain of hiPSCs-derived T lymphocytes by cDNA sequencing (Table 1). Twenty of twenty two randomly selected clones contained productive V(D)J rearrangements, and all 20 displayed different DNA sequences in the V(D)J junctions. Several different J segments were combined with different V_β_ sequences. These data demonstrate that the T cells generated from hiPSCs in our *in vitro* system undergo extensive TCR V(D)J recombination and nucleotide addition.

**Table 1:** Highly diverse CDR3 regions in populations of T cells derived in vitro from hiPSC.

V_β_	(D) region	J_β_	V_β_	J_β_
**CTCGGCCGTGTATCTCTGTGCCAGCAGC**	ACAGGG	TACAATGAGCAGTTCTTCGGGCCAGGGACAC	V11-2	J2-1
**CTCGGCCGTGTATCTCTGTGCCAGCAGCT**	ACA	CCTACAATGGGCAGTTCTTCGGGCCAGGGACAC	V11-2	J2-1
**CTCGGCCGTGTATCTCTGTGCCAGCAGCTTAGA**	GG	ATGAAAAACTGTTTTTTGGCAGTGGAACCCAG	V11-2	J1-4
**CTCGGCCGTGTATCTCTGTGCCAGCAGCTTA**	CAGGGGGCG	GAAAAACTGTTTTTTGGCAGTGGAACCCAG	V11-2	J1-4
**CTCGGCCGTGTATCTCTGTGCCAGCAGC**	CAGGG	TGAAAAACTGTTTTTTGGCAGTGGAACCCAG	V11-2	J1-4
**CTCGGCCGTGTATCTCTGTGCCAGCAGCTT**	CCA	TAATGAAAAACTGTTTTTTGGCAGTGGAACCCAG	V11-2	J1-4
**CTCGGCCGTGTATCTCTGTGCCAGCAGCT**	*	CCTACGAGCAGTACTTCGGGCCGGGCACCAGG	V11-2	J2-7
**CTCGGCCGTGTATCTCTGTGCCAGCAGCTTAG**	GG	TACGAGCAGTACTTCGGGCCGGGCACCAGG	V11-2	J2-7
**CTCGGCCGTGTATCTCTGTGCCAGCAGCTTAGACA**	GGGG	AGAGACCCAGTACTTCGGGCCAGGCACGCG	V11-2	J2-5
**CTCGGCCGTGTATCTCTGTGCCAGCAGCT**	GGACAGG	AGAGACCCAGTACTTCGGGCCAGGCACGCG	V11-2	J2-5
**CTCGGCCGTGTATCTCTGTGCCAGCAGCT**	ACAGGG	AAGAGACCCAGTACTTCGGGCCAGGCACGCG	V11-2	J2-5
**CTCGGCCGTGTATCTCTGTGCCAGCAGCTTA**	CAGGGGGCG	AACTATGGCTACACCTTCGGTTCGGGGACCAGGT	V11-2	J1-2
**TTGGGGACTCGGCCATGTATCTCTGTGCCAGCAGC**	CAGGG	TGAAAAACTGTTTTTTGGCAGTGGAACCCAG	V11-1	J1-4
**GGACTCGGCCGTGTATCTCTGTGCCAGCAGCTTA**	ACTAGCGGGGGGGC	CACAGATACGCAGTATTTTGGCCCAGGCACCCGGC	V7-2	J2-3
**GGACTCGGCCGTGTATCTCTGTGCCAGCAGCTTAG**	GACAGGG	CTATGGCTACACCTTCGGTTCGGGGACCAGGT	V7-2	J1-2
**AGATTCGGCAGCTTATTTCTGTGCCAGCTCACCAC**	AGG	ACTATGGCTACACCTTCGGTTCGGGGACCAGGT	V18	J1-2
**CACATACCTCTCAGTACCTCTGTGCCAGCAGTGAA**	CAGGGGG	ACACTGAAGCTTTCTTTGGACAAGGCACCAGA	V25-1	J1-1
**CACATACCTCTCAGTACCTCTGTGCCAGCAGTGA**	CAACA	TGAACACTGAAGCTTTCTTTGGACAAGGCACCAGA	V25-1	J1-1
**GGAGGACTCAGCCATGTACTTCTGTGCCAGCAGTG**	G	GAACACTGAAGCTTTCTTTGGACAAGGCACCAGA	V2	J1-1
**GACAGCTTTCTATCTCTGTGCCAGTAGTATAGACA**	*	GCTCCTACAATGAGCAGTTCTTCGGGCCAGGGACAC	V19	J2-1

Sequences of 20 TCR-β cDNA clones. Productive rearrangements were detected for 20 of 22 randomly selected clones.

Sequences between Vβ and Jβ were designated D regions. *Clones in which no Vβ and Jβ sequences were detected around the V(D)J junction region.

**Figure 4:**
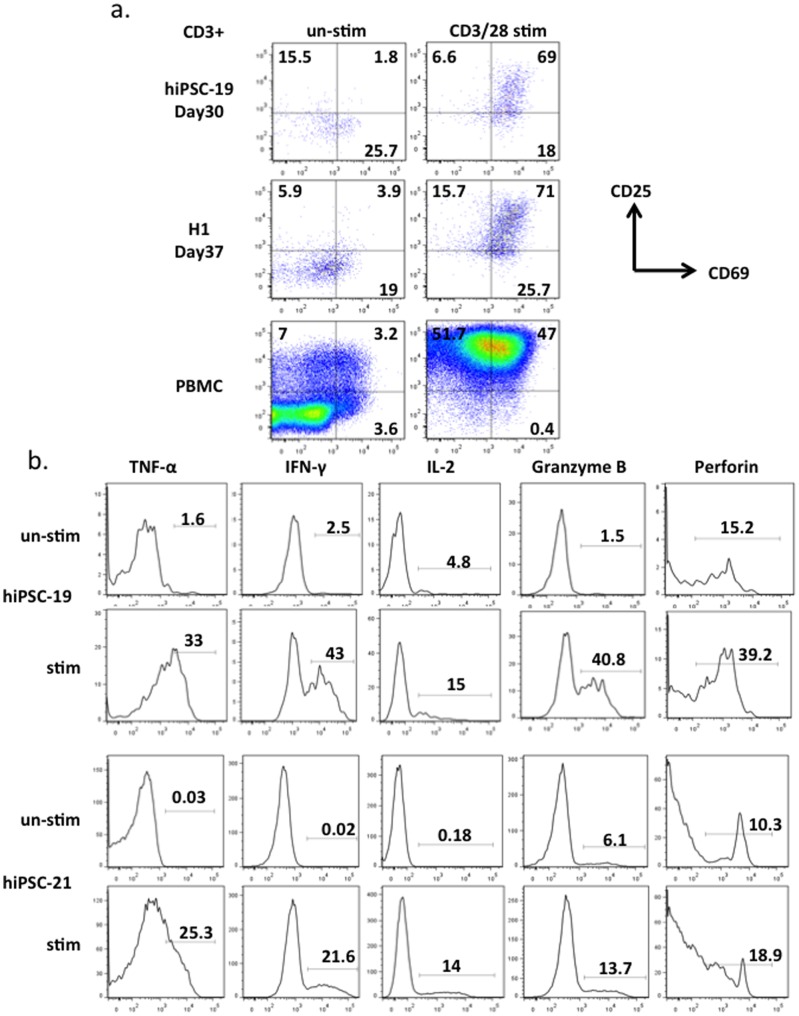
T cell functional assays. **a**, Day30 T cells derived from hiPSC-19, day 37 T cells derived from H1 and human PBMC primary T cells were stimulated with anti-CD3/28 beads for 3 days before FACS analysis of activation markers CD25 and CD69. The data were gated on CD3+ populations. **b**, Day29 T cells derived from hiPSC-19 and hiPSC-21 were stimulated by PMA and ionomycin for 12 hrs before analysis of the intracellular proteins TNF-α, IFN-γ, IL-2, Granzyme-B, and Perforin. Cells were gated on CD3+ and CD56- populations.

Finally, we examined whether T cells derived from hiPSCs and hESCs *in vitro* are functional. We stimulated these T cells (Figure 4) and primary human T cells (Figure S13) with different reagents that are known to activate T cells isolated from human peripheral blood and quantitated surface marker expression and cytokine production. After culture with anti-CD3/CD28 beads, CD25 and CD69 double positive T cells derived from hiPSCs increased from 2% to 69%; double positive cells derived from hESCs increased from 4% to 71% (Figure 4a). These T cells also up-regulated the expression of IL-2, IFN-γ and TNF-α after stimulation with PMA and ionomycin (Figure 4b). Granzyme-B and Perforin, which are secreted by both cytotoxic T cells and NK cells, were also observed in CD3+ and CD56- populations after mitogen activation. These data suggest that T cells generated by our system will be capable of killing specific target cells if engineered with the appropriate recombinant TCRs [32], [33].

## Discussion

In summary, we have successfully produced populations of αβ and γδ T lymphocytes with a broad TCR repertoire from human iPSCs *in vitro*. During differentiation for 35 days in culture, these cells express markers indicative of normal development. At the end of the differentiation protocol, the cells can be induced with PMA and ionomycin to produce IL-2, IFN-γ, TNF-α, Granzyme-B and Perforin. These responses are all indications of normal T lymphocyte function.

One human ESC line (H1) and four human iPSC lines reprogrammed from one healthy skin donor were used in this study to generate mature T lymphocytes. All lines successfully generated CD3+, CD4+ and CD8+ T cells and NK cells (CD56+) in all experiments. Although a wide deviation in mean fluorescence intensity (MFI) of CD4 antibody staining was observed in our data, the same deviation was also observed when primary human CD34+ cells isolated from mobilized peripheral blood were differentiated in our system. Therefore, this CD4+ staining pattern does not appear to be intrinsic to iPSC-derived T lymphocytes but may reflect differences in *in vitro* and *in vivo* differentiation conditions.

Interestingly, we observe few mature CD4+CD8- cells in our system. At the end of our T cell differentiation culture *in vitro*, CD4−CD8+ and CD4+CD8+ T lymphocytes predominate. In vivo, DP cells interact with MHC class II (MHCII) proteins expressed by thymic epithelium, and this interaction stimulates maturation to CD4+CD8−. The requirement of MHCII for normal maturation is confirmed by the severe reduction in CD4+CD8− T lymphocytes in MHCII knockout mice [34]. Also, expression of other factors, such as ThPOK [35], TOX [36], GATA-3 [37], and RUNX [38] that participate in the determination of CD4 versus CD8 lineage specification may be influenced by interactions with thymic epithelium. Several groups have demonstrated that mouse T cell development *in vitro* is stimulated by thymic organ culture or culture with re-aggregated thymic epithelium [10], [39], [40]. In future experiments, perhaps immature T cells derived from hiPSC can be cultured with human thymic epithelium to generate both mature CD4+CD8− and CD4−CD8+ T lymphocytes. Also, patient-specific MSC (mesenchymal stem cell) cultures with and without recombinant DL-4 may substitute for OP-9 stromal cells to provide xeno-free conditions for production of hematopoietic progenitors and mature T lymphocytes.

Finally, genetic modification of patient-specific hiPSC followed by differentiation to mature T lymphocytes may provide new treatment options for patients with inherited or acquired immune deficiencies. Also, the addition of chimeric antigen receptor sequences (CARs) [32], [33] to patient-specific hiPSC may provide a method to produce virtually unlimited numbers of T lymphocytes designed to target specific tumor types. The insertion of CAR sequences at a single chromosomal location would enhance safety and provide specificity to this powerful approach to personalized cancer immunotherapy.

## Supporting Information

Figure S1 **Characterization of human induced pluripotent stem cells. a**, Primary human keratinocytes (PHK) derived from skin biopsy were reprogrammed to iPSC with a modified version of our ‘hit and run’ vector and subsequently infected with Adeno-Cre to remove the vector. PCR primers specific to lentiviral DNA were used to determine whether polycistronic reprogramming factor sequences in individual hiPSC clones were successfully deleted. PCR primers for endogenous genomic DNA were used as controls. **b**, hiPSCs growing in the plates were tested for alkaline phosphatase expression. **c**, Primary human keratinocytes (PHK), hESC (H1), and reprogramming factor-free hiPSC lines(hiPSC-19 and hiPSC-21) were examined for expression of pluripotent markers (KLF4, NANOG, OCT4, SALL4, SOX2, ZFP42, and UTF1) by nCounter analysis (NanoString Technologies). Expression levels of each gene in H1 cells were set to 1, and gene expression levels in PHK and hiPSCs were compared to H1. **d**, Teratomas were formed by injecting hiPSC-19 and hiPSC-21 cells into the dorsal flanks of NSG mice. The tumors were removed after 8 to 12 weeks and histological sections demonstrated tissues derived from all three germ layers.
(TIFF)

Figure S2 **In vitro derivation of CD34+ HSC/HPC from Human ESC.** Day11 and Day18 cells were derived from hESC-H1 as described in the text and analyzed by FACS.
(TIFF)

Figure S3 **Flow cytometric assay of CD2, CD7 and CD34 expression in T cells derived in vitro from hiPSC and hESC H1.** CD34+ cells were affinity purified from Day18 cultures as described in the text and cultured on OP9-DL4. Expression of (**a**) CD7 and CD34, (**b**) CD7 and CD2 was analyzed by FACS after 7, 14, 21, 28 and 35 days of co-culture.
(TIFF)

Figure S4 **Flow cytometric assay of CD1a and CD7 expression in T cells derived in vitro from hiPSCs (hiPSC-21).** CD34+ cells were affinity purified from Day18 cultures as described in the text and cultured on OP9-DL4. Expression of CD1a and CD7 was analyzed by FACS after 14, 21, 28 and 35 days of co-culture.
(TIFF)

Figure S5 **Flow cytometric assay of CD1a and CD7 expression in T cells derived in vitro from hiPSCs (hiPS-19).** CD34+ cells were affinity purified from Day18 cultures as described in the text and cultured on OP9-DL4. Expression of CD1a and CD7 was analyzed by FACS after 14, 21, 28 and 35 days of co-culture.
(TIFF)

Figure S6 **Flow cytometric assay of CD1a and CD7 expression in T cells derived in vitro from hESCs (H1).** CD34+ cells were affinity purified from Day18 cultures as described in the text and cultured on OP9-DL4. Expression of CD1a and CD7 was analyzed by FACS after 14, 21, 28, 35 and 42 days of co-culture.
(TIFF)

Figure S7 **Flow cytometric assay of CD3cyt, CD3 and CD56 expression expression in T cells derived in vitro from hiPSCs.** CD34+ cells were affinity purified from Day18 cultures as described in the text and cultured on OP9-DL4. Expression of (**a**) cytoplasmic CD3 (CD3cyt), (**b**) CD56 and surface CD3 was analyzed by FACS after 14, 21, 28 and 35 days of co-culture.
(TIFF)

Figure S8 **Generation of T lymphocytes from hiPSCs.** CD34+ cells were affinity purified from Day18 cultures as described in the text and cultured on OP9-DL4. Expression of CD4 and CD8 was analyzed by FACS after 7, 14, 21, 28 and 35 days of co-culture.
(TIFF)

Figure S9 **In vitro differentiate primary human CD34+ cells to T cells.** Human mobilized peripheral blood CD34+ cells were cultured on OP9-DL4. Expression of CD4 and CD8 was analyzed by FACS after 46 days of co-culture.
(TIFF)

Figure S10 **Flow cytometric assay TCR-γδ and TCR-αβ expression in T cells derived in vitro from hiPSC-19.** CD34+ cells were affinity purified from Day18 cultures as described in the text and cultured on OP9-DL4. Expression of (**a**) TCR-γδ and surface CD3, **(b)** TCR-αβ and surface CD3 was analyzed by FACS after 14, 21, 28 and 35 days of co-culture.
(TIFF)

Figure S11 **Flow cytometric assay TCR-γδ and TCR-αβ expression in T cells derived in vitro from hESC-H1.** CD34+ cells were affinity purified from Day18 cultures as described in the text and cultured on OP9-DL4. Expression of (**a**) TCR-γδ and surface CD3, **(b)** TCR-αβ and surface CD3 was analyzed by FACS after 14, 21, 28 and 35 days of co-culture.
(TIFF)

Figure S12 **Flow cytometric assay of TCR-Vβ in human primary peripheral T cells.** Human primary peripheral T cells were typed for TCRV_β_ with the Beta Mark TCR Repertoire Kit from Beckman Coulter. The kit contains eight vials. Each vial contains 3 monoclonal antibodies that recognize 3 different V_β_ chains. The antibodies were conjugated with FITC, PE or FITC plus PE. A total of 24 different mAbs in this kit can detect 24 V_β_ chains (belonging to 19 different families). The cells were gated on the CD3+ population.
(TIFF)

Figure S13 **Primary human T cell functional assays.** Human primary PBMC were stimulated by PMA and ionomycin for 12 hrs before analysis of the intracellular proteins TNF-α, IFN-γ, IL-2, Granzyme-B, and Perforin by FACS. Cells were gated on CD3+ and CD56- populations.
(TIFF)
