# Supplementary figures and images for: Broad T-Cell Receptor Repertoire in T-Lymphocytes Derived from Human Induced Pluripotent Stem Cells
Source: PLoS One. 2014 May 14;9(5):e97335. doi: 10.1371/journal.pone.0097335 (PMC4020825; doi:10.1371/journal.pone.0097335)

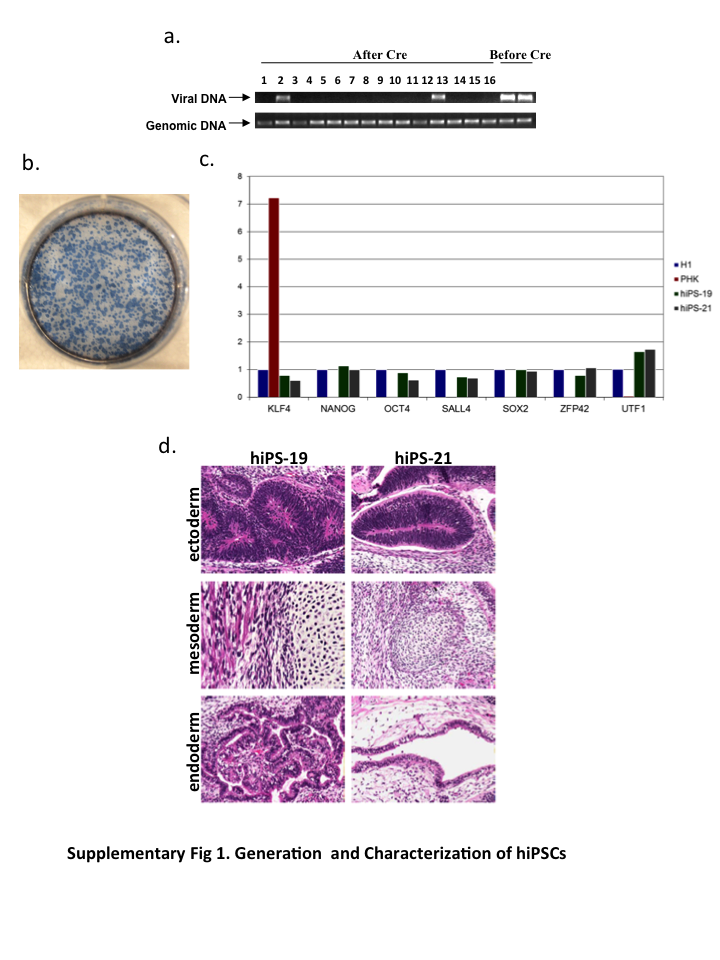

Supplement: Figure S1 — Characterization of human induced pluripotent stem cells. a, Primary human keratinocytes (PHK) derived from skin biopsy were reprogrammed to iPSC with a modified version of our ‘hit and run’ vector and subsequently infected with Adeno-Cre to remove the vector. PCR primers specific to lentiviral DNA were used to determine whether polycistronic reprogramming factor sequences in individual hiPSC clones were successfully deleted. PCR primers for endogenous genomic DNA were used as controls. b, hiPSCs growing in the plates were tested for alkaline phosphatase expression. c, Primary human keratinocytes (PHK), hESC (H1), and reprogramming factor-free hiPSC lines(hiPSC-19 and hiPSC-21) were examined for expression of pluripotent markers (KLF4, NANOG, OCT4, SALL4, SOX2, ZFP42, and UTF1) by nCounter analysis (NanoString Technologies). Expression levels of each gene in H1 cells were set to 1, and gene expression levels in PHK and hiPSCs were compared to H1. d, Teratomas were formed by injecting hiPSC-19 and hiPSC-21 cells into the dorsal flanks of NSG mice. The tumors were removed after 8 to 12 weeks and histological sections demonstrated tissues derived from all three germ layers. (TIFF) [file pone.0097335.s001.tiff]

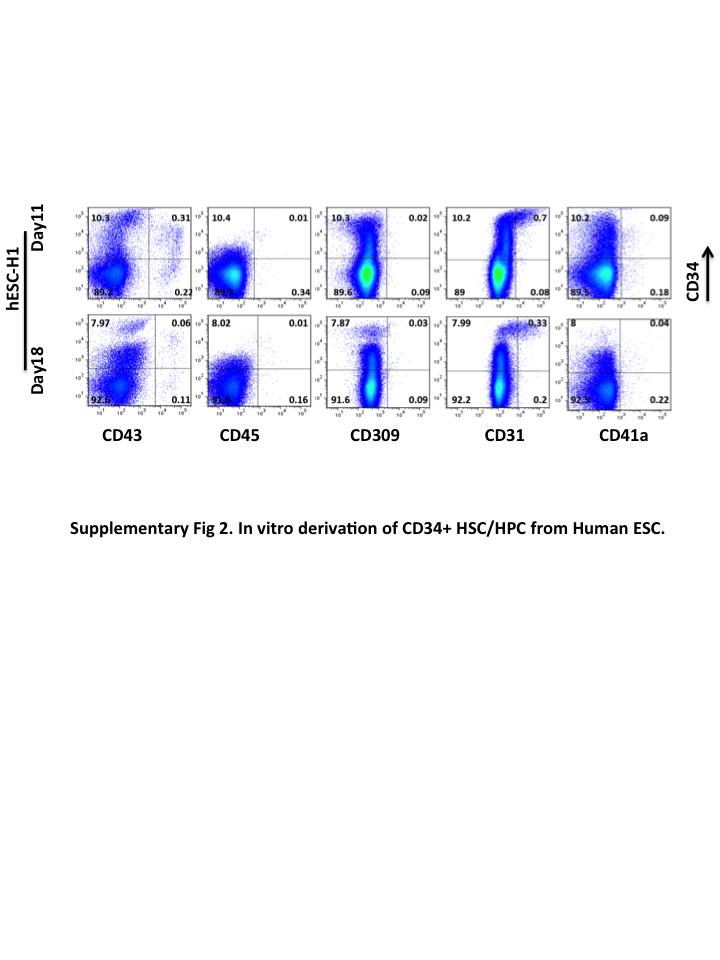

Supplement: Figure S2 — In vitro derivation of CD34+ HSC/HPC from Human ESC. Day11 and Day18 cells were derived from hESC-H1 as described in the text and analyzed by FACS. (TIFF) [file pone.0097335.s002.tiff]

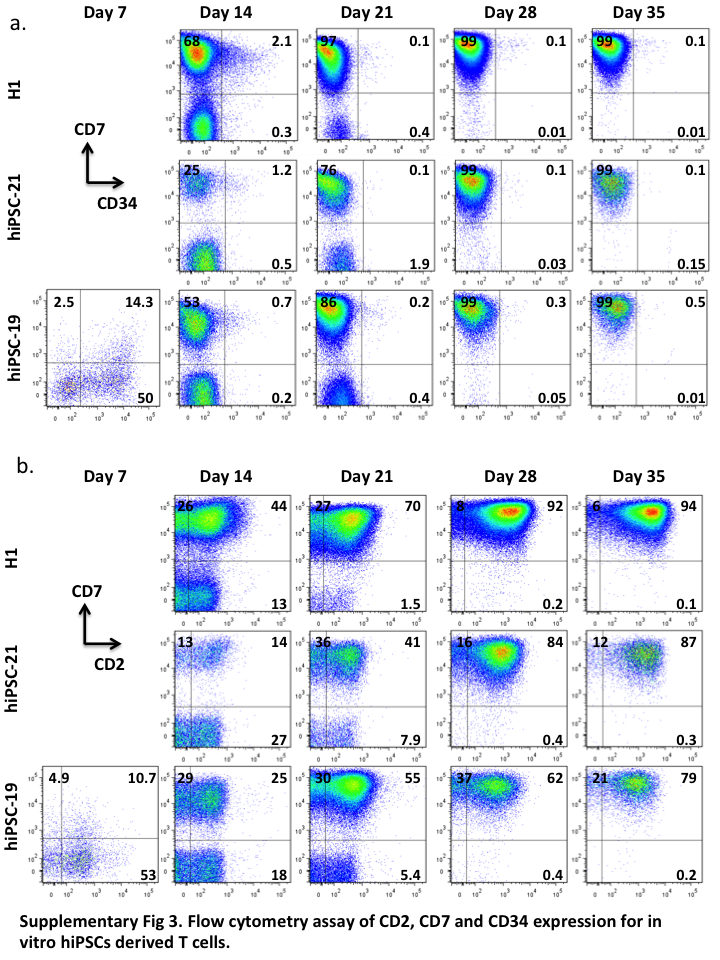

Supplement: Figure S3 — Flow cytometric assay of CD2, CD7 and CD34 expression in T cells derived in vitro from hiPSC and hESC H1. CD34+ cells were affinity purified from Day18 cultures as described in the text and cultured on OP9-DL4. Expression of (a) CD7 and CD34, (b) CD7 and CD2 was analyzed by FACS after 7, 14, 21, 28 and 35 days of co-culture. (TIFF) [file pone.0097335.s003.tiff]

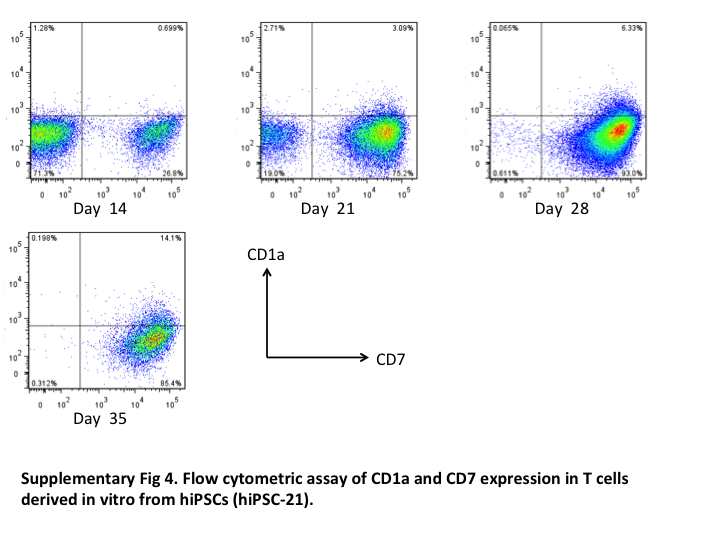

Supplement: Figure S4 — Flow cytometric assay of CD1a and CD7 expression in T cells derived in vitro from hiPSCs (hiPSC-21). CD34+ cells were affinity purified from Day18 cultures as described in the text and cultured on OP9-DL4. Expression of CD1a and CD7 was analyzed by FACS after 14, 21, 28 and 35 days of co-culture. (TIFF) [file pone.0097335.s004.tiff]

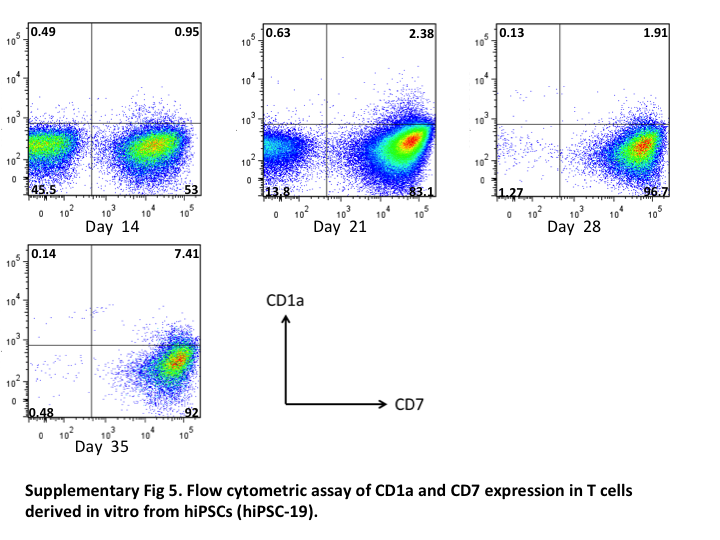

Supplement: Figure S5 — Flow cytometric assay of CD1a and CD7 expression in T cells derived in vitro from hiPSCs (hiPS-19). CD34+ cells were affinity purified from Day18 cultures as described in the text and cultured on OP9-DL4. Expression of CD1a and CD7 was analyzed by FACS after 14, 21, 28 and 35 days of co-culture. (TIFF) [file pone.0097335.s005.tiff]

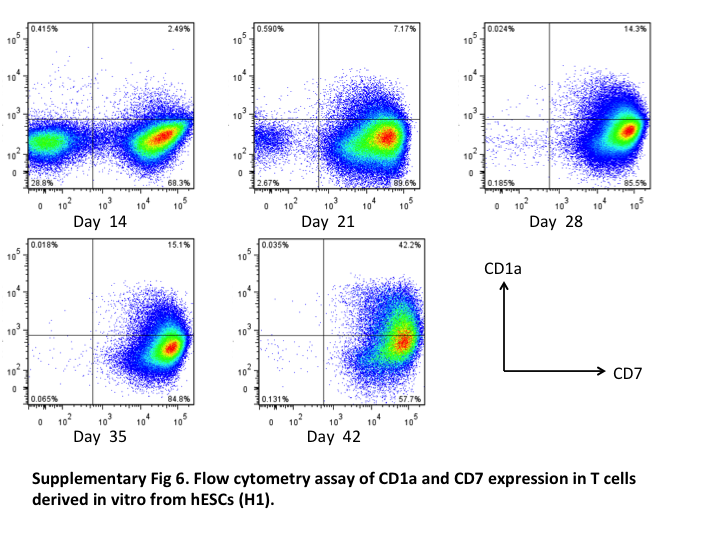

Supplement: Figure S6 — Flow cytometric assay of CD1a and CD7 expression in T cells derived in vitro from hESCs (H1). CD34+ cells were affinity purified from Day18 cultures as described in the text and cultured on OP9-DL4. Expression of CD1a and CD7 was analyzed by FACS after 14, 21, 28, 35 and 42 days of co-culture. (TIFF) [file pone.0097335.s006.tiff]

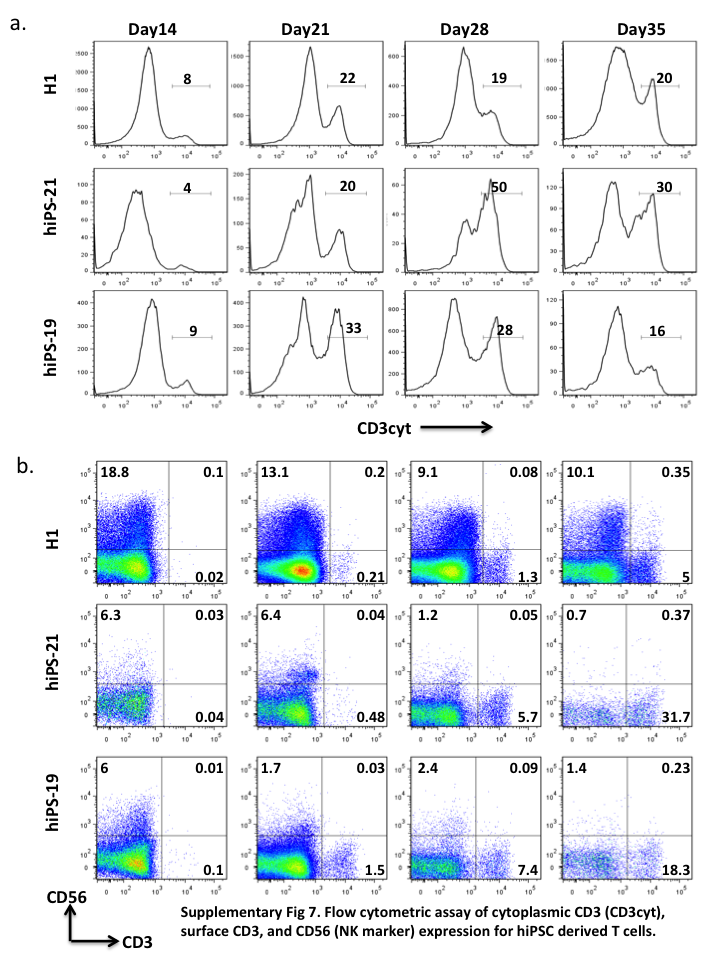

Supplement: Figure S7 — Flow cytometric assay of CD3cyt, CD3 and CD56 expression expression in T cells derived in vitro from hiPSCs. CD34+ cells were affinity purified from Day18 cultures as described in the text and cultured on OP9-DL4. Expression of (a) cytoplasmic CD3 (CD3cyt), (b) CD56 and surface CD3 was analyzed by FACS after 14, 21, 28 and 35 days of co-culture. (TIFF) [file pone.0097335.s007.tiff]

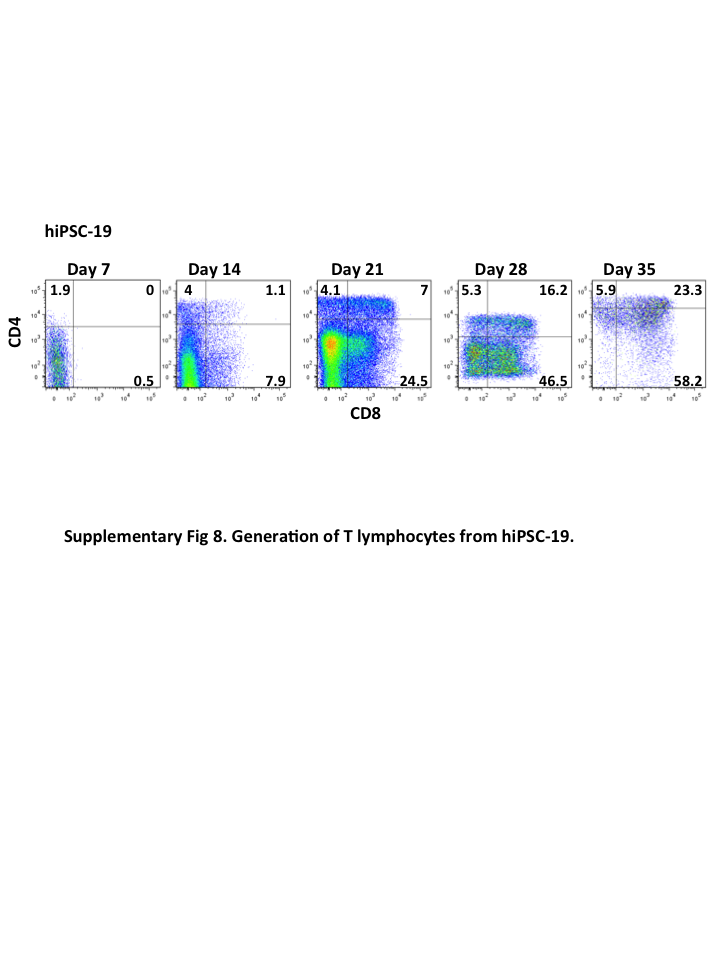

Supplement: Figure S8 — Generation of T lymphocytes from hiPSCs. CD34+ cells were affinity purified from Day18 cultures as described in the text and cultured on OP9-DL4. Expression of CD4 and CD8 was analyzed by FACS after 7, 14, 21, 28 and 35 days of co-culture. (TIFF) [file pone.0097335.s008.tiff]

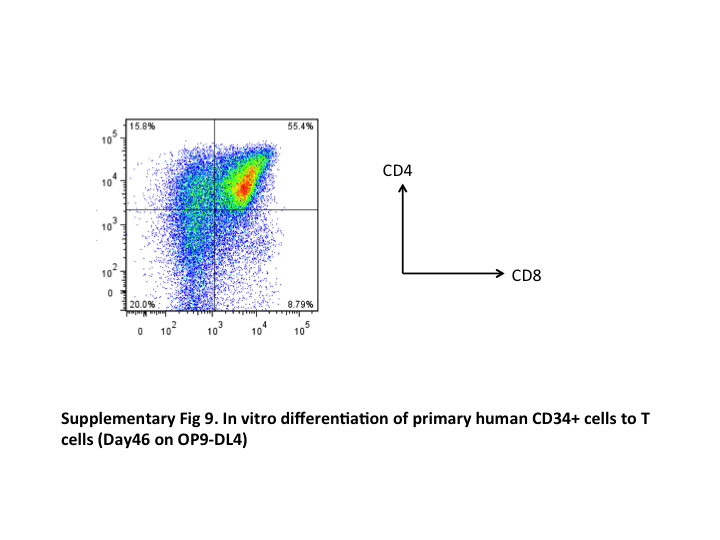

Supplement: Figure S9 — In vitro differentiate primary human CD34+ cells to T cells. Human mobilized peripheral blood CD34+ cells were cultured on OP9-DL4. Expression of CD4 and CD8 was analyzed by FACS after 46 days of co-culture. (TIFF) [file pone.0097335.s009.tiff]

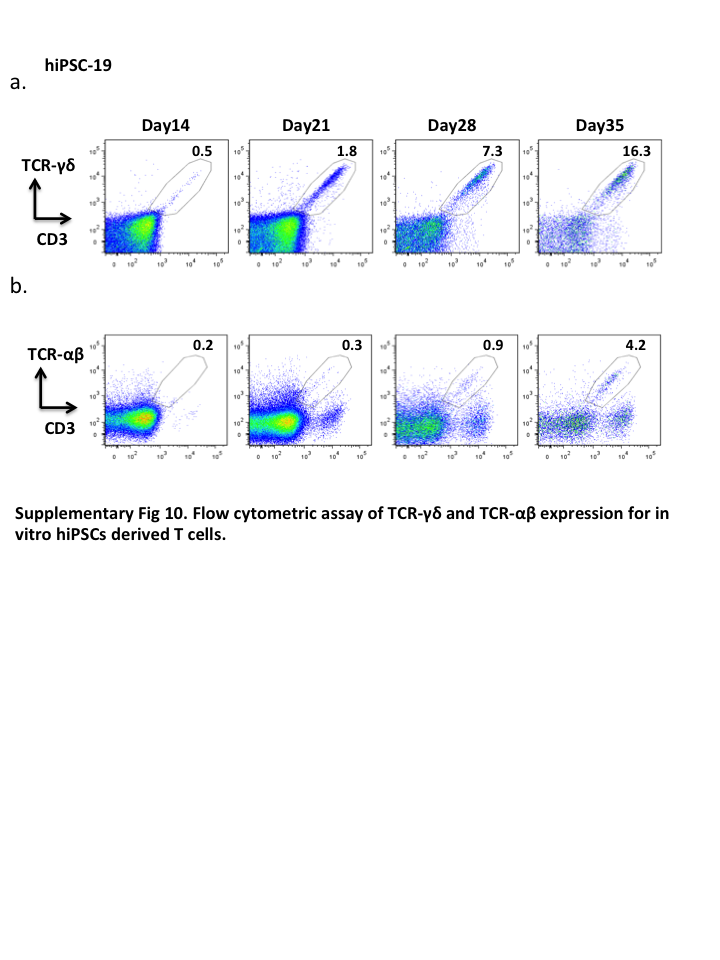

Supplement: Figure S10 — Flow cytometric assay TCR-γδ and TCR-αβ expression in T cells derived in vitro from hiPSC-19. CD34+ cells were affinity purified from Day18 cultures as described in the text and cultured on OP9-DL4. Expression of (a) TCR-γδ and surface CD3, (b) TCR-αβ and surface CD3 was analyzed by FACS after 14, 21, 28 and 35 days of co-culture. (TIFF) [file pone.0097335.s010.tiff]

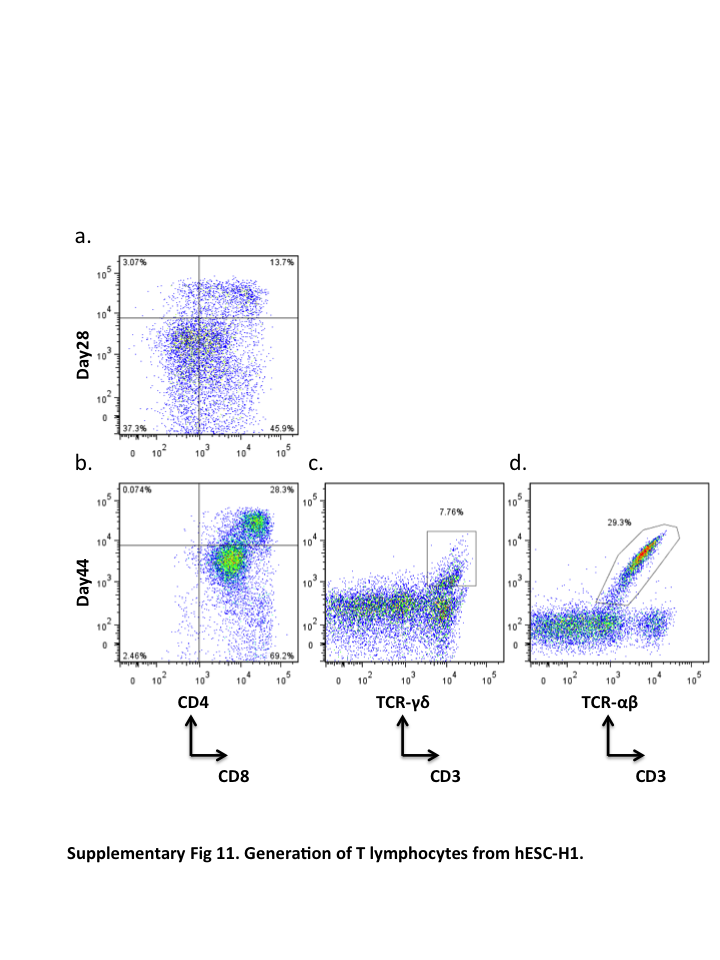

Supplement: Figure S11 — Flow cytometric assay TCR-γδ and TCR-αβ expression in T cells derived in vitro from hESC-H1. CD34+ cells were affinity purified from Day18 cultures as described in the text and cultured on OP9-DL4. Expression of (a) TCR-γδ and surface CD3, (b) TCR-αβ and surface CD3 was analyzed by FACS after 14, 21, 28 and 35 days of co-culture. (TIFF) [file pone.0097335.s011.tiff]

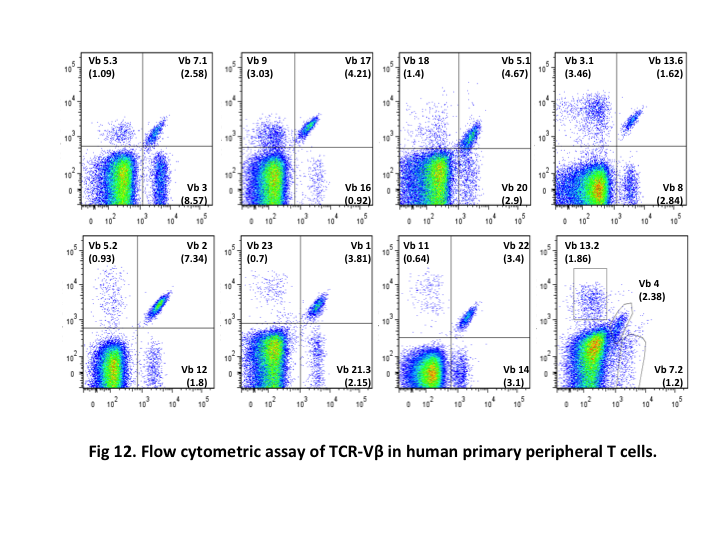

Supplement: Figure S12 — Flow cytometric assay of TCR-Vβ in human primary peripheral T cells. Human primary peripheral T cells were typed for TCRVβ with the Beta Mark TCR Repertoire Kit from Beckman Coulter. The kit contains eight vials. Each vial contains 3 monoclonal antibodies that recognize 3 different Vβ chains. The antibodies were conjugated with FITC, PE or FITC plus PE. A total of 24 different mAbs in this kit can detect 24 Vβ chains (belonging to 19 different families). The cells were gated on the CD3+ population. (TIFF) [file pone.0097335.s012.tiff]
